# Investigation of rumen long noncoding RNA before and after weaning in cattle

**DOI:** 10.1186/s12864-022-08758-4

**Published:** 2022-07-22

**Authors:** Alexis Marceau, Yahui Gao, Ransom L. Baldwin, Cong-jun Li, Jicai Jiang, George E. Liu, Li Ma

**Affiliations:** 1 Department of Animal and Avian Sciences, University of Maryland, 8127 Regents Drive, MD 20742 College Park, USA; 2 Animal Genomics and Improvement Laboratory, USDA-ARS, Building 306, BARC-East, MD 20705 Beltsville, USA; 3 Department of Animal Science, North Carolina State University, 27695 Raleigh, USA

## Abstract

**Background:**

This study aimed to identify long non-coding RNA (lncRNA) from the rumen tissue in dairy cattle, explore their features including expression and conservation levels, and reveal potential links between lncRNA and complex traits that may indicate important functional impacts of rumen lncRNA during the transition to the weaning period.

**Results:**

A total of six cattle rumen samples were taken with three replicates from before and after weaning periods, respectively. Total RNAs were extracted and sequenced with lncRNA discovered based on size, coding potential, sequence homology, and known protein domains. As a result, 404 and 234 rumen lncRNAs were identified before and after weaning, respectively. However, only nine of them were shared under two conditions, with 395 lncRNAs found only in pre-weaning tissues and 225 only in post-weaning samples. Interestingly, none of the nine common lncRNAs were differentially expressed between the two weaning conditions. LncRNA averaged shorter length, lower expression, and lower conservation scores than the genome overall, which is consistent with general lncRNA characteristics. By integrating rumen lncRNA before and after weaning with large-scale GWAS results in cattle, we reported significant enrichment of both pre- and after-weaning lncRNA with traits of economic importance including production, reproduction, health, and body conformation phenotypes.

**Conclusions:**

The majority of rumen lncRNAs are uniquely expressed in one of the two weaning conditions, indicating a functional role of lncRNA in rumen development and transition of weaning. Notably, both pre- and post-weaning lncRNA showed significant enrichment with a variety of complex traits in dairy cattle, suggesting the importance of rumen lncRNA for cattle performance in the adult stage. These relationships should be further investigated to better understand the specific roles lncRNAs are playing in rumen development and cow performance.

**Supplementary Information:**

The online version contains supplementary material available at 10.1186/s12864-022-08758-4.

## Background

For many years, genomic research has focused on the direct line of genes to gene products to phenotypic observations. Recent advances in sequencing technologies have led to more in depth exploration of the genomic regions that are transcribed into RNA but rarely into a protein product. Connections have been established between these non-coding RNAs and regulation of gene expression in many organisms. Examples include the X chromosome inactivation, allelic imprinting, pluripotency control, cancer, and many other biological processes [1]. A subset of these noncoding RNAs are the long noncoding RNA (lncRNA): these transcripts are at least 200 nucleotides in length, with some reaching up to 32,000 nucleotides, and are found across nearly all species [2]. Previous research has identified large numbers of lncRNA across many model and non-model species, ranging from > 7,000 transcripts in cattle [3] to over 270,000 lncRNA in humans [4]. Recent research in cattle has revealed more lncRNA transcripts in many tissues across cattle breeds: over 4,000 in 6 different tissues between 2 Chinese cattle breeds, nearly 10,000 in 18 different bovine tissues, over 23,000 lncRNA in bovine testes tissue as they mature, and 1,535 lncRNAs in bovine oocytes [5–7]. Additionally, almost 8,000 lncRNAs were found to be associated with metabolic efficiency [8].

A key characteristic of the cow is the four-part stomach system, where feed enters the reticulum and rumen, passes through the omasum, and reaches the abomasum where digestion occurs in a manner similar to humans and other livestock animals. Many organs comprise a small proportion of body as the animal matures, but the rumen increases from 30 to 70% of the capacity of the gut during weaning and continues to grow throughout lactation [9, 10]. However, at birth, a calf has only fully developed the abomasum. The fermentation vat including the reticulum and rumen is sterile upon birth and takes several weeks to establish a bacterial colony suitable for ruminant digestion. Therefore, new calves are mostly fed with milk or a milk replacer that can use an esophageal groove to bypass the under-developed digestion system, although dry feed is required to develop rumen bacterial activity. The anaerobic nature of the under-developed rumen, when dry feed is added, makes a vessel suitable for anaerobic bacterial growth, producing acetic, propionic, and butyric acids, lowering the pH, of the rumen and continuing bacterial growth [11]. As the rumen develops post-natal, the bacterial colonies grow and genetic/genomic changes likely occur to aid in the transition from a milk-based, pre-weaning diet to the feed or grass diet after weaning. It has been shown that rumen development and rumen microbiomes are affected by the weaning process across different weaning strategies [12].

It has been well documented that gene expression changes have implications in rumen pH maintenance, gastrointestinal tract cell proliferation, growth, and development [13]. LncRNA has been shown to have regulatory roles of gene expression. In humans, approximately 5% of the genome is conserved solely due to genomic regions with regulatory roles, with 80% of these regions being associated with chromatin state, adding more evidence to the hypothesis that lncRNAs are regulatory elements [14]. Given the recent increase in interest in non-coding genomic regions, it would not be surprising to find connections between these non-coding RNAs and changes in gastrointestinal behaviors, such as weaning [15].

Previous research has shown that the weaning process does lead to changes in gene expression in rumen. Butyrate can promote rumen development though assisting with structural integrity, epigenetic regulation, signaling pathways, and more [16]. As butyrate enters the rumen via milk or top-dressed feeds, 1,977 genes were identified as differentially expressed with or without butyrate treatment in rumen epithelial tissue — these genes included many key regulators and signaling pathways [17]. Weaning also led to variability in the chromatin structures and extracellular interactions in rumen tissues. The shift from milk to feed led to epigenetic changes including DNA methylation, histone modification, and more [18]. Lin et al. provided increasing evidence that rumen development hinges on changes in genetic interactions, both expression-wise and extracellularly [17]. The previously identified 6,679 novel lncRNA found in rumen tissue gives additional support [19]. As lncRNAs are associated with pre-transcriptional regulation, transcriptional regulation, post-transcriptional regulation, as well as acting as signaling molecules, decoys, scaffolds, and guides, it is expected to find lncRNAs functioning in the rumen tissue [20]. The previous research also lends credence to the hypothesis that the two weaning conditional rumen samples will show differential expression levels in lncRNA.

Given the regulatory roles associated with lncRNA, analysis of lncRNA and their difference before and after weaning in rumen may reveal important regulatory pathways that allow a calf to be adequately weaned and begin life with a healthy digestive system. To further explore the features and potential functions of lncRNA in cattle, here we report a genome-wide study by isolating and investigating RNA transcripts that meet coding and length criteria for lncRNA classification, using rumen epithelial tissue in calves before and after weaning. With industrial tools, samples were sequenced and mapped to a robust genome before being filtered based on size, coding status, coding potential, and similarities to known genes (Supplemental Figure 1). The resulting transcript lists were then analyzed for differential expression, sequence conservation, multi-species orthologs, and enrichment with GWAS results of 42 dairy cattle traits that involve production, reproduction, body conformation, and health components. These findings shed light on the lncRNA landscape within cattle and their rumen, allowing us to further unravel the genetic mechanisms at work for tissue development and dairy cattle performance.

## Results

### LncRNA identification

To identify lncRNA, Illumina HiSeq 2500 (PE150) sequencing was performed on three pre-weaning rumen samples and three post-weaning samples in Holstein cattle. We generated a total of over 125,000,000 sequence reads, averaging 21,085,595 reads per sample. Double-iterative mapping via Hisat yielded approximately 87.97% alignment in each sample [21]. Stringtie and Stringtie-merge yielded a consensus sequence for pre-weaning samples with 312,432 fragments and 315,559 fragments in the post-weaning samples [22, 23].

**Fig. 1:**
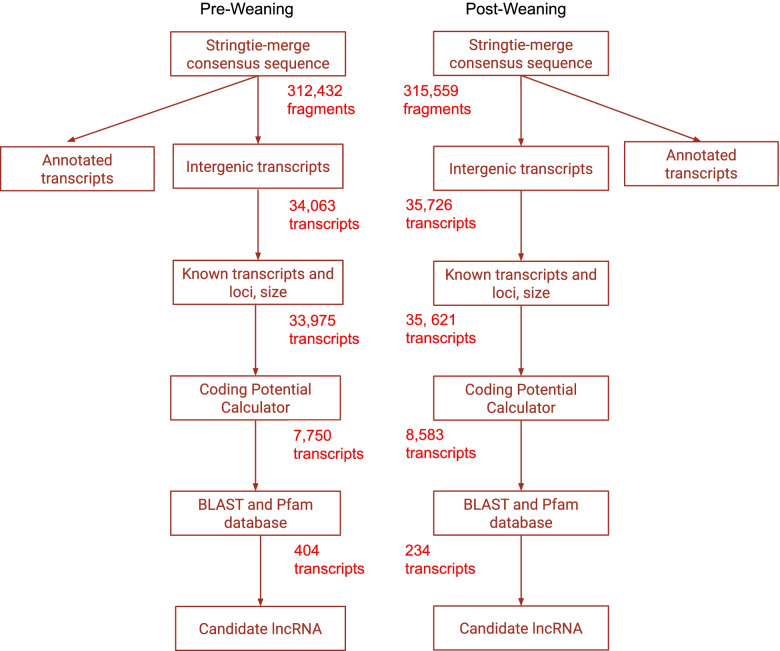
Filtering of Transcripts in Pre-weaning and Post-Weaning Rumen Tissue Samples. Once a consensus sequence was generated for each sample, a series of filtering steps were used to isolate candidate lncRNA. Steps included removing known protein coding transcripts, removing transcripts possessing coding potential, and those that demonstrated nucleotide and protein sequence homology. In the pre-weaning rumen tissue sample, 404 transcripts remained, and 234 transcripts remained in the post-weaning rumen tissue sample

Comparing consensus sequences to the current ARS-UCD 1.2 *Bos taurus* reference genomes annotated transcripts based on known loci and genes [24]. Results from the comparisons allowed for filtering based on overlapping with known loci and transcripts in both reference genomes that resulted in 33,975 remaining transcripts in the pre-weaning samples and 35,621 transcripts in the post-weaning samples, respectively. These transcripts were then analyzed with a coding potential calculator, a BLAST search, and comparisons to the Pfam database to remove any remaining coding transcripts [25–27]. Finally, filtered results produced a list of 404 candidate lncRNA transcripts in the pre-weaning tissue and 234 transcripts in the post-weaning tissue (Fig. 1).

### LncRNA characteristics with comparison to protein coding transcripts

**Fig. 2:**
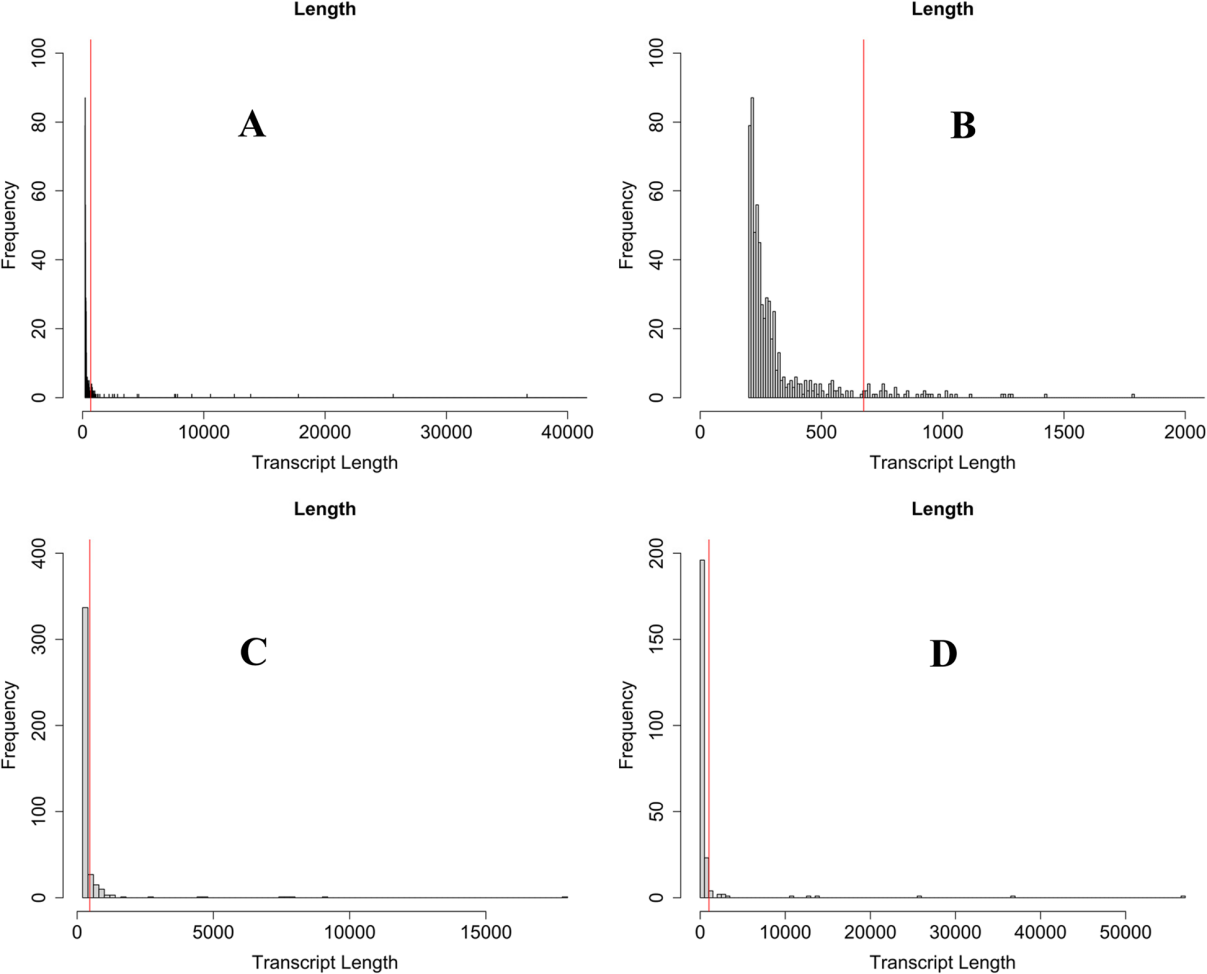
Length distribution of candidate lncRNA transcripts. **A** Length of all candidate lncRNA transcript. The average length of transcripts measured 674 base pairs, indicated by red line. **B** Zoomed in distribution of length of all candidate lncRNA transcript. Excluding those longer than 2000 base pairs for added clarity.  **C** Length of pre-weaning transcripts, ranging from 200 to 17809 and averaging 466 nucleotides. **D** Length of post-weaning transcripts, ranging from 200 to 56626 and averaging 1033 nucleotides

Based on the ARS-UCD1.2 *Bos taurus* annotation [24], size of gene transcript ranged from 200 to over a million nucleotides, averaging 28,239 nucleotides. This is in stark contrast to the length of lncRNA transcripts we identified in rumen, which averaged 674 nucleotides overall; pre-weaning lncRNAs averaged 466 nucleotides and post-weaning averaged 1,033 nucleotides (Fig. 2). Although all identified lncRNAs were over 200 nucleotides in length, as required by the definition, they averaged 674 nucleotides and ranged from 200 to ~18,000 base pairs in pre-weaning samples, and 200-57,000 base pairs in post-weaning samples (Fig. 2). This is in line with previous findings of lncRNA transcripts being shorter than their coding counterparts. All identified lncRNA had, on average, 1.01 exons per transcript, whereas the NCBI gene annotation had 1-335 exons per transcript with an average of 9.2 exons per transcript. A distinctly lower number of exons per transcript in lncRNA compared to coding transcripts appears to be common across many studies [14, 28].

**Fig. 3:**
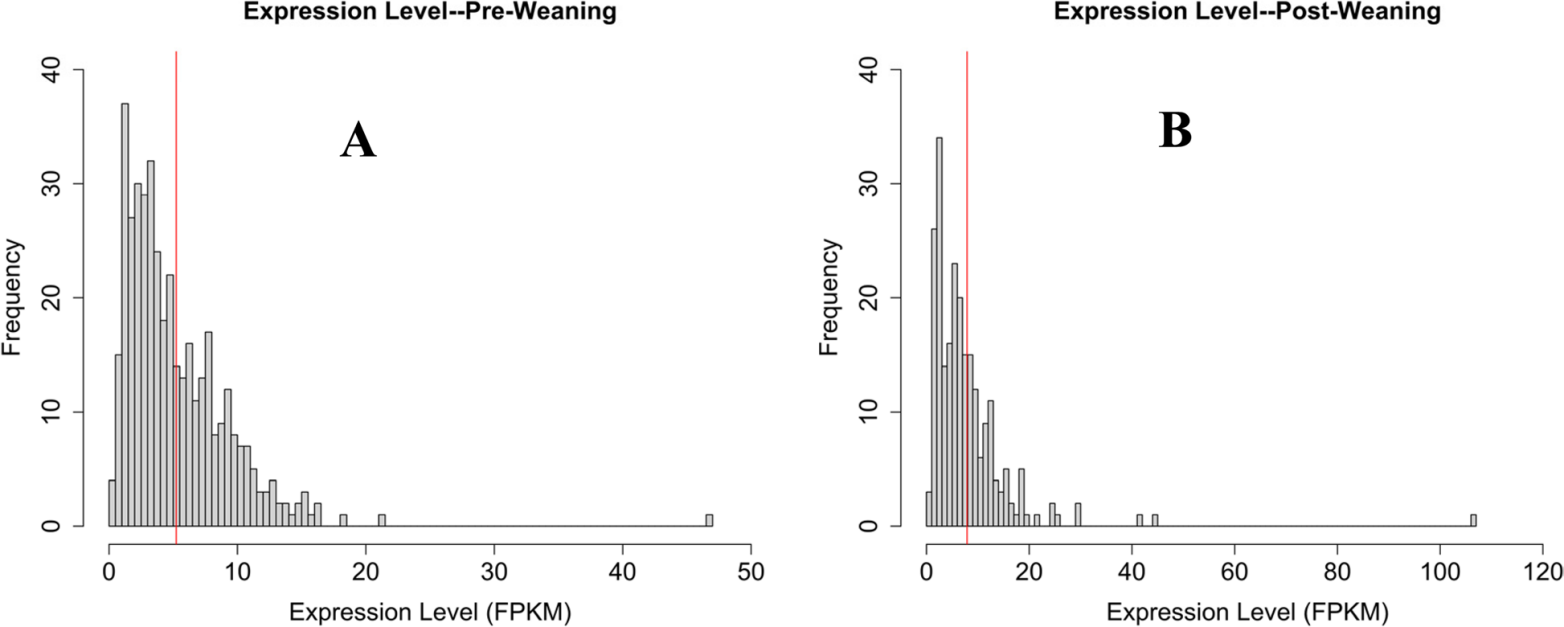
Expression of lncRNA candidate transcripts. **A** FPKM values of transcripts expressed in pre-weaning tissue. Expression levels ranged from 0.17 to 46.81 FPKM, averaging 5.24 FPKM. The average length of transcripts was indicated by red line. **B** FPKM values of transcripts expressed in post-weaning tissue. Expression levels ranged from 0.72 to 106 FPKM, averaging 7.89 FPKM. The average length of transcripts was indicated by red line

In pre-weaning cattle, expression level of coding genes averaged 26.12 fragments per kilobase of transcript per million mapped reads (FPKM). This is five times more than the lncRNA identified in pre-weaning samples, which measured an average of 5.24 FPKM. In post-weaning samples, lncRNA expression averaged an FPKM value of 7.89, compared to the 27.89 FPKM average value of all gene transcripts (Fig. 3). The lower expression level of lncRNA compared to coding genes is also consistent with previous studies.

### Differential expression analysis before and after weaning

The expression level of rumen lncRNA was similar before and after weaning with an average of 5.24 and 7.89 FPKM pre- and post-weaning, respectively. When analyzing the lncRNA profiles of pre- and post-weaning rumen tissue, the majority of lncRNAs identified were present only in one condition, with 395 found only in pre-weaning tissues and 225 only in post-weaning samples. Still, nine transcripts were present under both weaning conditions. However, none of these common transcripts showed expression levels that varied at a statistically significant level (Table 1).

**Table 1:** T-test of expression levels between pre and post weaning rumen tissue for nine common lncRNA transcripts. Transcripts were isolated as common if they overlapped with each other, average expression was calculated for each transcript and is reported in FPKM. A paired student t-test was performed with degrees of freedom equaling 8. This yielded a critical value of 1.860, which none of the t-test scores surpassed, indicating none of the lncRNAs identified as common to both conditions were differentially expressed at a significant level

Pre-weaning		Post-weaning		*P*-value
**lncRNA**	**Expression**	**lncRNA**	**Expression**	
Chr1:143606037–143,606,970	1.167	Chr1:143606047–143,607,059	2.963	0.743
Chr1:146868890–146,870,164	0.798	Chr1:146868985–146,869,483	2.004	0.798
Chr20:23927430–23,927,631	46.81	Chr20:23927420–23,927,884	106.4	0.584
Chr4:119469477–119,469,730	3.01	Chr4:119469677–119,470,016	1.776	0.514
Chr5:118054620–118,054,855	0.722	Chr5:118054288–118,054,860	1.607	0.654
Chr7:46217152–46,217,456	3.399	Chr7:46217122–46,217,472	5.292	0.515
Chr7:12950022–12,950,226	13.54	Chr7:12950002–12,950,242	11.11	0.464
Chr7:36273737–36,274,435	1.494	Chr7:36273748–36,274,421	1.348	0.471
Chr7:43944991–43,945,389	1.430	Chr7:43944829–43,945,374	1.757	0.516

### Analysis of lncRNA sequence conservation

LncRNA tends to show lower rates of sequence conservation when compared to coding genes. To investigate this in cattle, transcript profiles for all coding transcripts, intergenic transcripts, and lncRNAs for both pre- and post-weaning conditions were converted to the human genome equivalents using the LiftOver v3.13 software and run through the PhastCons program to calculate conservation scores among transcripts of 46 vertebrates [29].

**Fig. 4:**
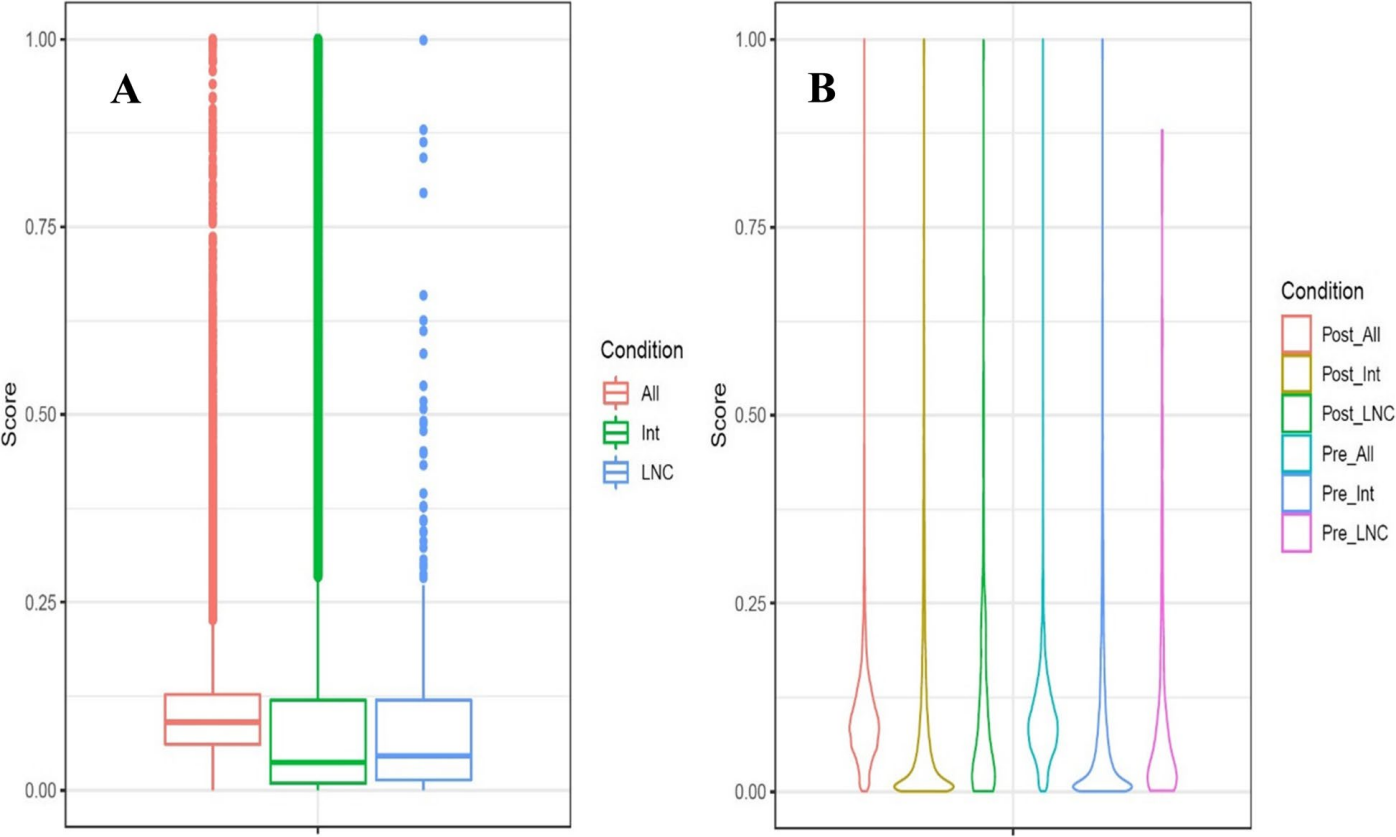
Phastcons scores of pre- and post-weaning conditions at whole genome, intergenic region, and lncRNA levels. **A** Boxplot of PhastCons scores for all transcripts, all intergenic transcripts, and all lncRNA candidate transcripts. **B** Violin plot of all six profiles: all preweaning transcripts, preweaning intergenic regions, preweaning lncRNA transcripts, all postweaning transcripts, postweaning intergenic regions, and postweaning lncRNA transcripts

**Fig. 5:**
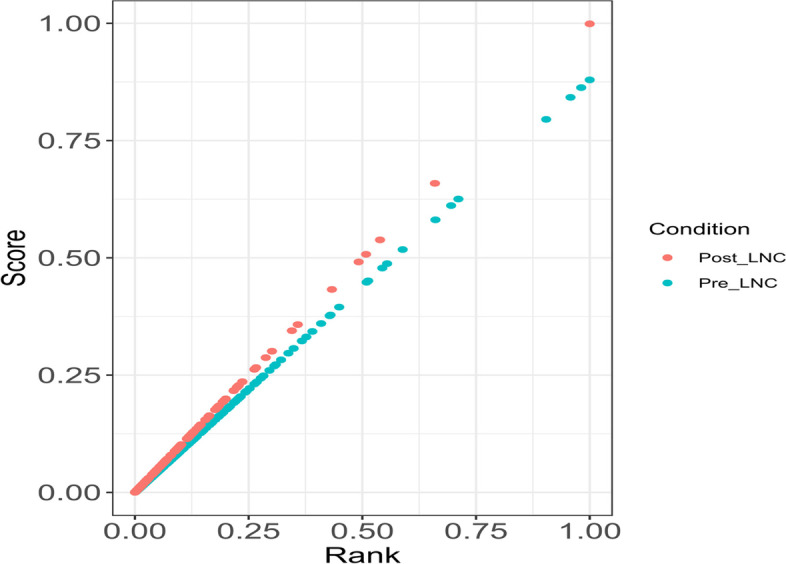
Scatter plot of lncRNA PhastCons scores. Most lncRNAs show scores well below 0.50 with a small number being well conserved across many species. Pre-weaning scores ranged from 0.000873 to 0.879405, and post-weaning scores ranged from 0.000183 to 0.658853, with an outlier of 0.98854

As expected, whole genome gene profiles showed higher scores overall than both intergenic and lncRNA profiles (Fig. 4). Interestingly, the median conversation score was slightly higher in lncRNA than intergenic transcripts. Although when plotted as a violin graph, the scores of lncRNA are localized near 0, intergenic regions are slightly more conserved. All transcripts averaged a PhastCons score of 0.103, intergenic regions scored an average of 0.104, and lncRNA had the lowest average score of 0.100. Complete profiles ranged in scores from 0.000111 to 0.999982, intergenic profiles ranged from 0 to 1, and lncRNA scores were as small as 0.000183 to as large as 0.998854. Between pre- and post-weaning, conservation scores were very similar (Fig. 4B). Pre-weaning samples ranged from 0.000873 to 0.879405, averaging 0.09963. Although it should be noted the median score of conserved pre-weaning transcripts was 0.0434. In post-weaning transcripts, scores ranged from 0.000183 to 0.98854 with an average score of 0.101 and a median score of 0.0485. LncRNA PhastCons scores were plotted based on score rank to illustrate clustering patterns (Fig. 5). Most lncRNAs showed low sequence conservation scores with a small number showing much higher scores. These findings are consistent with lncRNA trends regarding conservation. The conservation score of the nine common transcripts were also calculated, which ranged from 0.008296 to 0.161162 with an average score of 0.0477 and a median of 0.0261.

### Transcriptional annotation of common and conserved lncRNA

In addition to the nine common transcripts in two weaning conditions, the top 10% of lncRNAs based on conservation scores were also isolated from both weaning profiles for further analyses, in an attempt to ascertain lncRNA function. Twenty-three transcripts were kept from the pre-weaning profile, and 14 transcripts were kept for the post-weaning profile. Using the UCSC genome browser, these 46 transcripts were compared to the human and mouse genomes to identify previously annotated genes. Of the nine common lncRNA transcripts, three matched to human and mouse genes: *ESYT2*, *SEC24A*, and *ZFN491*/*Zfp811*. A fourth lncRNA matched to the *Clic6* gene in *Rattus norvegicus*. Functions of these genes include ion channel formation, cellular lipid transport, transport vesicle formation, transcriptional activity, and more. Eleven of the 23 top conserved pre-weaning lncRNA showed matches to human and/or mouse genes. These include *NLGN1*, *SAP130*, *ATP13A2*/*Padi3*, *BCL1*, *NTNG1*, *SLC2A1*, *APBB2*, *OAZ1*, *MEX3D*, *ANXA6*, and *DOCK11*. Roles include nervous system development, cell surface protein interactions, transcriptional repressors, signal transduction, neuronal circuit formation, glucose transportation, post-transcriptional regulation, and much more. Of the post-weaning transcripts, nine of the 14 candidates matched to human/mouse genes: *4930448K20Rik*, *LAPTM4A*, *MBD5*, *IGSF21*, *F11R*, *EPS15*, *RAB21*, *COL25A1*, *UHRF1*, and *PLK5*. Roles include nucleoside transport, heterochromatin binding, synapse inhibition, membrane traffic control, fibrillization inhibition, epigenetic regulation, cell cycle progression, and a number of other roles. Most notably, the *F11R* gene plays a role in epithelial tight junction formation; given the rumen is lined with stratified squamous epithelial cells, this has interesting implications in the relationship between lncRNA and rumen development [30]. Another interesting finding is the *EPS15* gene, which has associations with cell growth regulation [31]. Given the physical changes taking place as the weaning process progresses, cell growth is a key component of the rumen maturation. The large number of roles these equivalent genes play fit with previous research of the vast number of functions lncRNAs exhibit in the biological systems.

### SNP heritability Enrichment analysis on cattle traits

By integrating 404 pre- and 234 post-weaning lncRNAs with large-scale GWAS data in Holstein cattle [32, 33], we revealed an interesting relationship between rumen lncRNA and cattle complex traits of economic importance. Using a method for partitioning SNP heritability named MPH (https://github.com/jiang18/mph), we quantified SNP heritability enrichment as the ratio of per-SNP heritability near the lncRNAs to the genome-wide one. *P*-value was also computed by comparing the enrichment level to 1 using a Wald test. Finally, our enrichment analysis used 7,988 and 4,856 variants within the lncRNA identified for pre-weaning and post-weaning conditions, respectively.

As a result, we found a significant enrichment of per-SNP heritability near the lncRNAs under pre and post weaning conditions across cattle production, reproduction, health, and body conformation traits (Table 2). Overall, post-weaning lncRNAs showed slightly more significant enrichment with cattle traits than pre-weaning lncRNAs, indicating a more important function of the rumen lncRNAs after weaning for cow performance in the adult stage. Stature was highly significantly associated with lncRNAs in both pre- (11.67x; *P* = 5.7E-6) and post-weaning (25.01x; *P* = 1.1E-9) conditions, reflecting the important functions of rumen lncRNAs under both pre and post weaning conditions related to the overall tissue development and growth. Livability was associated with lncRNAs under both weaning conditions (11.34x; *P* = 0.01 for pre and 15.1x; *P* = 0.01 for post, respectively), suggesting that rumen development may have a functional role in the regulation of immunity and disease resistance. Milk’s SNP heritability was only significantly enriched in post-weaning lncRNAs (5.38x; *P* = 0.04). And daughter pregnancy rate (DPR) was only significantly enriched with post-weaning lncRNAs (14.9; *P* = 6.4E-4).

**Table 2:** Enrichment of rumen lncRNA in cattle GWAS results. Traits analyzed for SNP heritability enrichment included cattle production (milk), reproduction (daughter pregnancy rate, DPR), health (livability), and body conformation (stature). Enrichment was analyzed in both pre and post weaning tissue conditions

	Milk			DPR			Stature			Livability		
	**Enrichment**	**SE**	**P**	**Enrichment**	**SE**	**P**	**Enrichment**	**SE**	**P**	**Enrichment**	**SE**	**P**
**Pre**	3.49	1.76	0.08	3.76	2.55	0.14	11.67	2.43	5.7E-06	11.34	4.46	0.01
**Post**	5.38	2.52	0.04	14.9	4.31	6.4E-04	25.01	4.02	1.1E-09	15.07	6.23	0.01

## Discussion

In this study, Illumina high throughput RNA-seq data were used to detect and analyze lncRNA in cattle rumen tissue before and after calf weaning. This was done to both identify rumen lncRNA and to find transcripts that are differentially expressed as the rumen develops from immature to mature. RNA-seq data were aligned to a robust reference genome and then progressively filtered by criteria such as coding potential, intergenic support, and size, resulting in a list of 629 lncRNA transcripts. Candidate transcripts averaged a shorter length, less exons per transcript, lower expression, and lower conservation scores when compared to whole genomic transcripts. This represents 404 transcripts in the pre-weaning profile, 234 transcripts in the post-weaning profile, and 9 transcripts common to both profiles. Interestingly, the 9 common transcripts are expressed at similar levels, indicating they likely play a basal role in rumen tissue that is independent of rumen tissue development.

This study confirms that there are lncRNA transcripts expressed in rumen tissue, and furthermore that transcripts are expressed differently as weaning progresses. Identification of hundreds of transcripts unique to before and after weaning conditions suggests the expression of these transcripts is related to the maturation of the animal, likely being tied to the weaning process. Overall, the variability in identified lncRNA transcripts indicates they likely play a role in the biological changes that occur as the calf’s digestive system develops. This also supports theories that although these transcripts are not made directly into gene product, they are essential for the growth and development of organisms. Enrichment analysis demonstrated that, although isolated from rumen tissue, these lncRNA transcripts likely influence traits outside of those associated with digestion/weaning, as several of the transcripts show significant enrichment based on genome wide association studies. A subset of the unique transcripts to each tissue condition showing enrichment in complex traits could mean many things, however, it is evidence that these transcripts are involved in the development of other characteristics as the calf is weaned. This could demonstrate that several transcripts may have broad applications in calf development and later performance overall.

When integrating the identified rumen lncRNA with existing GWAS results, we reported significant enrichments of GWAS signals in or near lncRNA regions across a wide spectrum of cattle traits, suggesting the importance of lncRNA on the genetics of complex traits. Considering the functional impact of lncRNA on gene regulation, future studies should no longer ignore lncRNA in GWAS and fine-mapping studies. In addition, genomic selection may also consider adding important SNPs near lncRNA onto newer SNP arrays and using different weights in the modelling process.

Previous studies have identified at least 7,000 lncRNA within cattle, as well as 20,000 + in other systems, and this study was able to identify over 600 transcripts that met lncRNA criteria when analyzing rumen tissue from *Bos taurus* [3, 4, 34]. Compared to other studies of cattle lncRNA, the current study is limited by only focusing on the rumen tissue and two weaning conditions. With only three replicates within each condition, we may not be able to discovery all the important lncRNAs in rumen due to limited detection power. Still, we emphasize the validity and importance of our results based on the evidence of consistent genomic features of the identified lncRNAs with existing studies, higher conservation across species than genome average, and enrichment with GWAS signals of important dairy cattle traits.

Further analyses of multiple tissues may also expand our findings and identify lncRNAs that are expressed exclusively in other tissues, as well as reveal those expressed in multiple tissues, including the rumen. Research has also shown lncRNA has connections with many biological processes such as chromosome X inactivation, allelic imprinting, pluripotency control, and cancer, so it is not surprising to find that lncRNA identified in *Bos taurus* rumen may be involved in development of the rumen, and likely across the organism [1]. This is supported by our findings that there are very different lncRNA profiles as a calf is weaning. Of both common and highly conserved lncRNAs, the corresponding genes in human and mouse models are indicative that these lncRNAs serve a number of roles related to rumen. Two of the identified genes are of particular interest: *F11R* and *EPS15*. *F11R* is related to the formation of the epithelial tight junctions, which is an interesting research endeavor given the rumen is lined with epithelial cells. The role of *EPS15* in cell growth regulation may also be particularly telling about the morphological changes that occur in the rumen as the calf is weaned. Although several genes show sequence similarities to known genes in well researched organisms, further research may shed light on the exact roles of these transcripts in either individual tissue or in a systematic setting.

## Conclusions

In this study, we investigated lncRNA features in cattle rumen tissue before and after weaning. As expected, cattle lncRNAs exhibited similar features as lncRNAs identified in other species. The majority of rumen lncRNAs are uniquely expressed in either the pre-weaning or post-weaning condition, indicating a functional role of lncRNAs in rumen development and transition of weaning. Overlapping with large-scale GWAS results in cattle, both pre- and post-weaning lncRNAs showed significant enrichment with all types of dairy traits of economic importance, suggesting the impact of rumen lncRNAs for cattle performance in the adult stage. The findings of this study allow for further research to be conducted to identify lncRNA in other tissues, investigate roles of candidate transcripts in traits of interest, and delve into lncRNA expression and developmental roles across an entire organism.

## Methods

### Animals and tissue Collection

 The animal handling procedures were approved by the Beltsville Area Animal Care and Use Committee Protocol Number 07–025. Six Holstein bull calves were used for the study, which were purchased from a local farm and transported by 3 days of age to the Beltsville Agricultural Research Center (BARC), Beltsville, MD, USA. All calves received colostrum at the first feeding and received standard diet afterward. We randomly selected three calves to be euthanized at 14 days of age for rumen epithelium collection to exemplify preweaning, and at 70 days of age, three calves were euthanized for the collection of rumen tissue postweaning, as previous research has demonstrated that a sample size of three is sufficient to find different gene expression related to organ development in dairy cattle [35].

The rumen tissues were collected from the ventral sac beneath the cranial pillar. Distinct visual cues present ensured the rumen tissue was predominately exposed to rumen liquor before slaughter. The collected tissues were rinsed with tap water and then rinsed in ice-cold physiological saline. According to manufacturer instructions, subsamples (~ 600 mg) of epithelial tissue were fixed in RNAlater RNA stabilization solution (Life Technologies, Grand Island, NY, USA) and stored at − 80 °C until RNA extraction.

### RNA sequencing, Transcriptional Mapping and Assembly

The RNA extraction procedure has been reported previously [36]. Briefly, total RNA was extracted by Trizol (Invitrogen, Carlsbad, CA, USA) followed by DNase digestion and Qiagen RNeasy column purification (Qiagen, Valencia, CA, USA). The RNA integrity was verified by an Agilent Bioanalyzer 2100 (Agilent, Palo Alto, CA, USA). After quality control procedures, total RNA was sequenced at 150-bp/paired-end sequence reads using RNA-sequencing service supplied by Novogene Corporation, Inc. (UC Davis Sequencing Center, Davis, CA, USA).

RNA sequence data of each sample were mapped to the ARS-UCD1.2 reference genome [24] using the spliced read aligner Hisat 2.2.1. A two-iteration approach was taken to capitalize on exon junction information [21]. The first iteration mapped samples to the reference using default settings. The second iteration leveraged junction findings from the first run to re-map reads to the ARS-UCD1.2 reference. Stringtie v2.1.4 was then used to assemble accepted hits into reports for each sample [23]. The ARS-UCD1.2 *Bos taurus* annotation was provided with the ‘–G’ option for more accurate assembly. All pre-weaning samples were merged using the ‘stringtie—merge’ function. The ARS-UCD1.2 annotation was provided again with the ‘–G’ option for accuracy. The above was performed again for the post-weaning samples. This provided a consensus sequence for both conditions for further analyses [23, 24].

### lncRNA identification

Identification of long non-coding RNAs (lncRNAs) started by using the CuffCompare 2.2.1 program. The ARS-UCD1.2 annotation fasta files were provided to improve classifications using the ‘-s’ option, and the ARS-UCD1.2 annotation .gtf files were provided as well via the ‘-r’ option to annotate any possible matching reference transcripts [37]. Any transcripts marked as overlapping with coding transcripts were removed to retain only intergenic transcripts. Remaining transcripts were inputted into the coding potential calculator (CPC), a software that scores reads based on open reading frame and homology analysis. Coding potential scores range from − 1 to 1, where a score below 0 indicates the transcript is likely non-coding (26). All transcripts that scored above 0 were removed. Those transcripts that remain were submitted through the CPC again to analyze the reverse compliment; again, any read scoring above 0 was removed. Next, transcripts were ran through the BLAST database to compare transcripts to known coding genes, removing any transcripts that were protein-coding but unannotated [25]. Transcripts were kept if the BLAST results showed no matching transcript. In order to remove any remaining non-lncRNA transcripts, reads were converted from bases to amino acids in all three reading frames before being compared to the Pfam database [26]. This removed any transcripts that showed documented protein domains. Reads that were less than 200 bp were also excluded, as they do not meet the lncRNA criteria. This produced a final list of transcripts for pre-weaning and post-weaning tissues that (a) did not overlap a known gene based on the NCBI and ARS-UCD1.2 reference genome, (b) were longer than 200 bp in length, (c) lacked coding potential, (d) did not show sequence similarity to any known gene, and (e) did not show any amino acid pattern resembling a known protein domain.

### Comparison to coding transcripts

Lengths were calculated for all transcripts in both the lncRNA and genome wide profiles under two weaning conditions. Profiles were compared to determine different patterns between subsets. Combined lncRNA and gene profiles were graphed as histograms to demonstrate contrast between profiles. The lncRNA length profiles were also graphed as a histogram with the average length calculated and plotted on the histogram using R 4.12 [38]. Additionally, a truncated graph was generated for clarity with average length denoted.

Using the Stringtie v2.1.4 software, expression levels for all transcripts in all samples were estimated. Stringtie v2.1.4 does so by clustering reads, creating a splice graph for each cluster that is associated with an identified transcript, creating a flow network for each transcript, then estimating expression level with a maximum flow algorithm [23]. Expression was reported with the fragments per kilobase of transcript per million mapped reads (FPKM) value. FPKM data for all reads were filtered into a subset containing only those reads in the final lncRNA list identified previously. Pre- and post-weaning expression levels were plotted in a histogram with the average value superimposed using R. Average expression of all transcripts was also calculated using the average command in Excel.

### PhastCons Analysis

PhastCons analysis was performed on lncRNA transcripts, full transcript profiles, and intergenic transcript profiles in both weaning conditions. This was done by using the UCSC LiftOver web tool to convert profiles from the ARS-UCD1.2 cattle genome to the hg38 human genome. The PhastCons command was then used on the open source galaxy server to compare genomic regions across 46 vertebrate species and the placental mammal subset of the vertebrate species to determine conservation scores (species include human, chimp, gorilla, orangutan, rhesus, baboon, marmoset, tarsier, mouse lemur, bushbaby, tree shrew, mouse, rat, kangaroo rat, guinea pig, ground squirrel, rabbit, pika, alpaca, dolphin, cow, horse, cat, dog, microbat, megabat, hedgehog, shrew, elephant, rock hyrax, tenrec, armadillo, sloth, wallaby, opossum, platypus, chicken, zebra finch, green anole, western clawed frog, tetraodon, fugu, stickleback, medaka, zebrafish, and lamprey). Each set was then graphed for comparisons. The top 10% of lncRNA transcripts based on PhastCons conservation score were extracted for further analyses.

### Transcriptional annotation

Candidate lncRNA transcripts were selected by either being present in both tissue conditions or by representing the top 10% of conserved sequences. The top 10% cutoff was selected as there was a clear separation of Phastcons scores between the top 10% and other transcripts. These regions were then searched in the UCSC genome browser to identify genes present in other species, primarily noting those found in humans and mice, as they represent well researched species. One transcript showed no matches to mice or humans but did match to a gene in *Rattus norvegicus*, and this was noted as well. Genes identified were then searched in UniProt to identify gene function.

### Heritability Enrichment Analysis

Heritability enrichment analysis was performed on two sets of lncRNA: those only present in pre-weaning and those in post-weaning rumen samples. GWAS data of ~ 27 K bulls and ~ 3 M SNPs/INDELspreviously reported [32, 33] were used for the enrichment analysis, representing cattle production (milk), reproduction (daughter pregnancy rate), health (livability), and body conformation (stature) phenotypes. The GWAS was performed in 27,214 Holstein bulls that had imputed 3 million SNPs and highly reliable predicted transmitting abilities (PTAs) for production, reproduction, health and body type traits.

We used a newly developed method, MPH, for the enrichment analysis (https://github.com/jiang18/mph).

In this analysis, MPH fits the following linear mixed model with two genetic components:

$$\begin{array}{ll}\boldsymbol{y}\;=\;\boldsymbol{X}\boldsymbol{b}\;+\;\boldsymbol{g}_1\;+\;\boldsymbol{g}_2\;+\;\boldsymbol{e}&\\\boldsymbol{g}_1\sim{N}(\boldsymbol{0},\boldsymbol{G}_1{\sigma}\;_{g1}^2\;)&\boldsymbol{e}\sim{N}(\boldsymbol{0},\boldsymbol{R}{\sigma}\;{}_e^2\;),\\\boldsymbol{g}_2\sim{N}(\boldsymbol{0},\boldsymbol{G}_2{\sigma}_{g2}^2\;)&\\&\end{array}$$

where **b** is a vector of fixed effects including intercept, **X** is a covariate matrix corresponding to **b**, **g**_1_ and **g**_2_ are two vectors of random effects representing the genetic contributions of the variants within lncRNAs and the remaining ones on the genome, respectively, and **e** is a vector of residual effects. **G**_1_ and **G**_2_ are two genomic relationship matrices constructed by genotypes corresponding to **g**_1_ (lncRNA) and **g**_2_ (remaining), respectively. We computed **G** using the second method as described in [39], $\varvec{G}=\frac{\varvec{Z}{\varvec{Z}}^{T}}{m}$, where **Z** represents standardized genotypes, and *m* is the number of variants in the GRM. We used *m*_1_ and *m*_2_ to represent the number of variants within lncRNAs and the number of the remaining ones on the genome, respectively. **R** is a diagonal matrix used to model individual reliability of deregressed PTAs. MPH uses a Monte Carlo REML algorithm similar to [40] to estimate variance components (VCs) and computes the variance-covariance matrix of VC estimates based on the Fisher information matrix.

The per-SNP genetic variance in lncRNA is equal to $\frac{{\sigma }_{{g}_{1}}^{2}}{{m}_{1}}$, and its per-SNP heritability enrichment is then computed by $\rho =\frac{\frac{{\sigma }_{{g}_{1}}^{2}}{{m}_{1}}}{\frac{({\sigma }_{{g}_{1}}^{2}+{\sigma }_{{g}_{2}}^{2})}{({m}_{1}+{m}_{2})}}$. We used the delta method to compute the standard error of *ρ*.

A total of 404 pre- and 234 post-weaning lncRNAs with 10 Kb window extension on both sides were used to test their enrichment of per-SNP heritability for four traits. We used a 10Kb window extension to cover linked regions considering the SNP density of our GWAS data and the linkage disequilibrium level in the Holstein cattle population [41–43]. The enrichment analysis included a total of 2,803,423 autosomal variants after QC with a minimal MAF of 0.005 and a minimal HWE p-value of 1E-8. We used 7,988 and 4,856 variants within the lncRNA identified for pre-weaning and post-weaning conditions, respectively. This analysis is achieved by partitioning SNP heritability with two genomic relationship matrices (GRMs): one made using SNPs/INDELs within lncRNAs and the other made using remaining ones. SNP heritability enrichment is quantified as the ratio of per-SNP heritability near lncRNAs to the genome-wide one, whose estimate and standard error are computed by MPH. Wald tests were used to compute *P*-value comparing an enrichment level to 1 (i.e., the genome-wide per-SNP heritability level).

## Supplementary Information

**Additional file 1: Supplemental Figure 1.** Pipeline of lncRNA identification. Pipeline design foridentification of lncRNA in pre-weaning and post-weaning rumen tissue samples.Color indicates before and after determining a consensus sequence for eachcondition.

## Data Availability

The datasets generated and analyzed during the current study are available in the NCBI SRA database (SUB8420017, BioProject ID: PRJNA672996). Analytical tools can be found at https://github.com/amarceau98/lncRNA-Identification-Pipeline.git.
