# Supplementary material for: Investigation of rumen long noncoding RNA before and after weaning in cattle
Source: BMC Genomics. 2022 Jul 22;23:531. doi: 10.1186/s12864-022-08758-4 (PMC9308236; doi:10.1186/s12864-022-08758-4)
Supplement: Supplementary file 1 — Additional file 1: Supplemental Figure 1. Pipeline of lncRNA identification. Pipeline design foridentification of lncRNA in pre-weaning and post-weaning rumen tissue samples.Color indicates before and after determining a consensus sequence for eachcondition. [file 12864_2022_8758_MOESM1_ESM.docx]

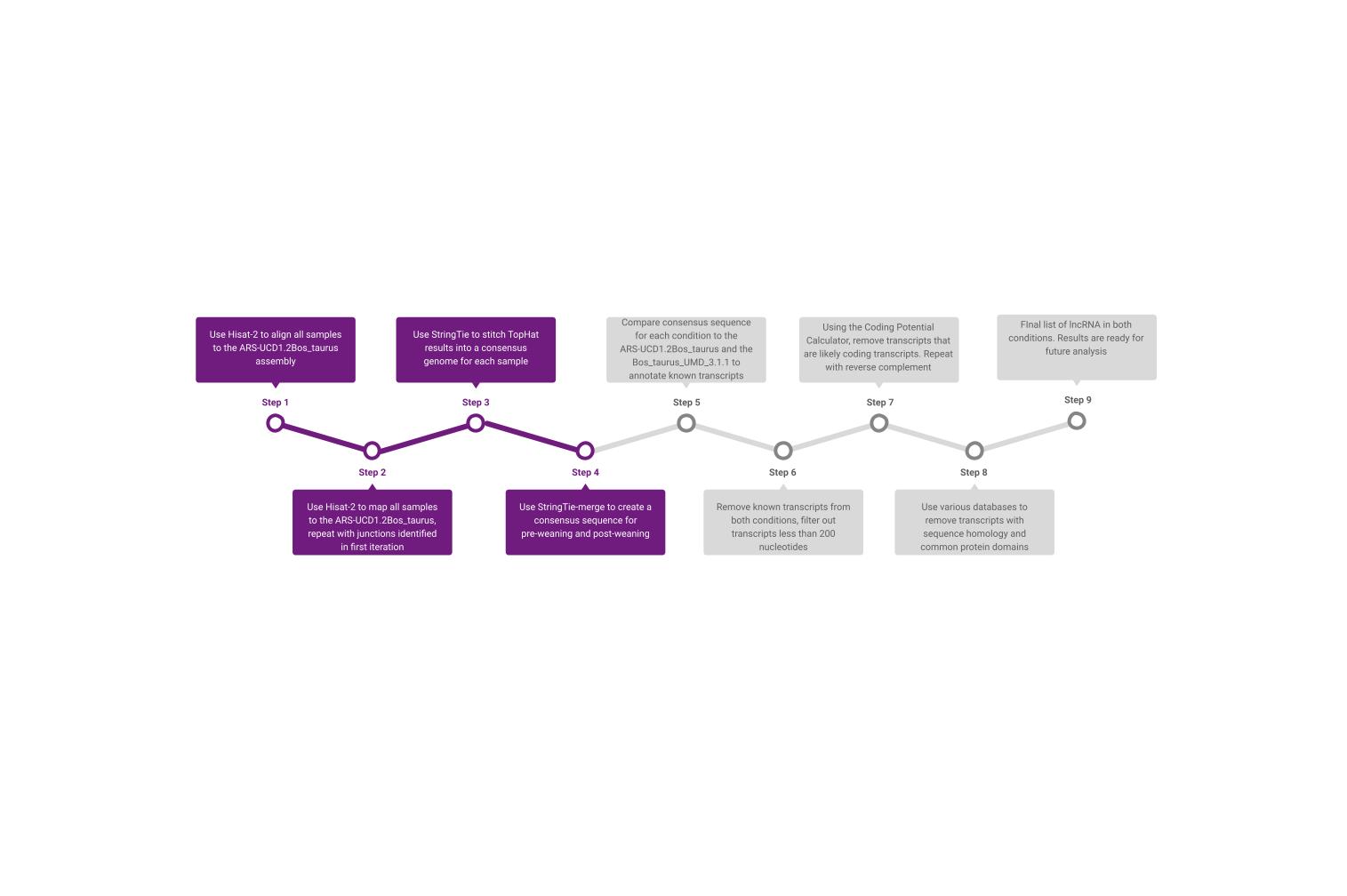


**Supplemental Figure 1: Pipeline of lncRNA identification**. Pipeline design for identification of lncRNA in pre-weaning and post-weaning rumen tissue samples. Color indicates before and after determining a consensus sequence for each condition.
